# Inhibition of miR-155 attenuates abdominal aortic aneurysm in mice by regulating macrophage-mediated inflammation

**DOI:** 10.1042/BSR20171432

**Published:** 2018-05-08

**Authors:** Zhidong Zhang, Kai Liang, Gangqiang Zou, Xiaosan Chen, Shuaitao Shi, Guoquan Wang, Kewei Zhang, Kun Li, Shuiting Zhai

**Affiliations:** 1 Department of Vascular and Endovascular Surgery, Henan Provincial People’s Hospital, NO.7 Weiwu Road, Zhengzhou, Henan 450003. P.R. China; 2 Fuwai Central China Cardiovascular Hospital, Beijing, China

**Keywords:** Abdominal aortic aneurysms (AAA), inflammation, miR155, macrophage

## Abstract

The aim of the present study was to identify abdominal aortic aneurysms (AAA)-associated miR-155 contributing to AAA pathology by regulating macrophage-mediated inflammation. Angiotensin II (AngII)–infused apolipoprotein E-deficient (ApoE-/-) mice and THP-1 cells model of miR-155 overexpression and deficiency were used in the experiments. The expression of miR-155 was detected by quantitative reverse transcription polymerase chain reaction (qRT-PCR). Cytokines were evaluated using enzyme-linked immunoabsorbent assay (ELISA). Western blotting was used to measure the levels of MMP-2, MMP-9, iNOS, and monocyte chemoattractant protein (MCP)-1 proteins. Immunostaining and transwell were used to determine CD68, elastic collagen, proliferation, and migration of vascular smooth muscle cells (VSMCs). The results showed that miR-155 and cytokines were up-regulated in AAA patients or ApoE-/- mice. Overexpression of miR-155 enhanced MMP-2, MMP-9, iNOS, and MCP-1 levels, and stimulated the proliferation and migration of VSMCs. Meanwhile, inhibition of miR-155 had the opposite effect. In addition, histology demonstrated accumulation of CD68 and elastic collagen-positive areas significantly decreased in miR-155 antagomir injection group. In conclusion, the results of the present study suggest that inhibiting miR-155 is crucial to prevent the development of AAA by regulating macrophage inflammation.

## Introduction

Abdominal aortic aneurysms (AAA) are defined as focal dilatation of the abdominal aorta when it is 50% greater than its normal diameter or when it is more than 3 cm in the abdominal aorta diameter. AAA is a common disease with an irreversible dilatation of the abdominal aorta, which is characterized by high mortality and asymptomatic [1]. Rupture is the most dreaded complication of AAA, resulting in approximate 90% mortality rates [1,2]. The progress of AAA is strongly associated with advanced age, sex, smoking, atherosclerotic disease and dyslipidemia, the presence of hypertension, and family history [3–6]. However, there are no treatment measures currently to effectively restrict AAA growth, and the mechanism of AAA remains unknown.

The present understanding characterizes AAA as a chronic inflammatory disease. Macrophages in AAA formation and rupture have significant effect [7]. Macrophages are localized in AAA lesions suggesting that they may play a key in the inflammatory cascade that precedes the disease [8]. The pathogenesis probably occurs with the injury of endothelial and vascular smooth muscle cells (VSMCs), which recruits an inflammatory response to lead to destroy the integrity of the vessel wall [9]. The inflammatory infiltration leads to a loss of the internal elastic lamina, myointimal hyperplasia, and ectopic distribution of VSMC in vascular disease [10]. Research have shown that phenotypic transition of VSMC drives the progression of vascular diseases, such as atherosclerosis, diabetes, restenosis, and hypertension [11]. VSMCs are associated with vessel injury and remodeling in the proinflammatory environment [12,13]. In the normal vessel wall, VSMCs are static, differentiated, contractile and have low rate of proliferation, and express high levels of contractile proteins such as α-smooth muscle actin (α-SMA) [14]. The injury of vascular wall causes a change in VSMC phenotype, which embodies as high expression of osteopontin (OPN) protein, then VSMC suffer to dedifferentiate, proliferate, and migrate into the vessel lumen [15]. In the last decade, there is increasing attention to identifying genetic that could perform more targeted screening for the mechanism of limiting AAA growth to help explore AAA pathogenesis [16–18].

The microRNAs (miRNAs) are small noncoding, double-stranded RNA molecules that can influence the stability of messenger RNAs (mRNAs) [19–21]. They are prominently expressed in many hematopoietic cell types and have emerged as potent regulators of vascular inflammation and cancer [22,23]. A recent study have highlighted the significance of miR-155 as regulatory elements of immune responses in various inflammatory transmitters [24]. It has been reported that miR155 level is increased in mouse and human with AAA, and there are a correlation between miR155 and proinflammatory cytokine under various conditions [25–27]. Macrophages are one of the critical cells of inflammation and immunology, which could serve as a mediator in the deterioration of AAA [28]. The existence of macrophage can weaken the arterial wall, because vessel collagens are disintegrated by MMP from macrophages [29]. However, the mechanisms by which macrophage may contribute to AAA pathogenesis remain undefined. Based on these premises, we analyzed that miR-155 mediates AAA formation *in vivo* and *vitro* by macrophages, and macrophages participate in the destruction of vascular structures and worsening of the inflammatory process in AAA.

## Materials and methods

### Clinical specimens

The biopsies and serum from patients undergoing elective open AAA repair (*n*=11, Age = 66 ± 5 years), and control subjects were selected from normal volunteer of the same age and sex without AAA (*n*=15, Age = 64 ± 6 years). Biopsies were obtained from the body of the AAA at the site of maximum AAA dilatation (AAA body) and from the macroscopically nondilated AAA neck (AAA neck), which were collected in Ambion® RNAlater® Tissue Collection (Thermo Fisher Scientific, Waltham, MA) and stored at −20°C. The AAA neck samples were used as controls, since previous studies suggest that aortic histology is relatively normal in these biopsies [30]. The blood samples from patients with AAA and normal volunteer. The blood samples were incubated for 30 min at 37°C, in water bath kettle to obtain serum or used to macrophage isolation. Patients with AAAs were defined as maximum aortic diameter ≥50 mm. The present study was approved by the Ethics Committee of Henan Provincial People’s Hospital and all patients provided informed consent.

### Model establishment and grouping

All animal experiments followed the guidelines of the Institutional Animal Care and Use Committee (IACUCs) of local. The AAA is defined as a 50% increase in external diameter of the abdominal aorta [31]. Eight-week-old male ApoE^−^/^−^ mice were provided by Shanghai Slac Laboratory Animal Co, Ltd. (Shanghai, China). Mice were divided into two groups, a group were used as AAA model (*n*=70) and another group turned into control (*n*=20). AAA was induced by chronic pour 800 ng/kg/min Angiotensin II (AngII) (Cat.no.9525, Sigma Aldrich, St. Louis, U.S.A.) into ApoE^−^/^−^ mice via mini-osmotic pumps (Model 1004, Alzet, CA, U.S.A.) for 28 days [32–35]. In another, two sets of ApoE^−^/^−^mice were infused with saline which served as control mice. After 28 days, 36 AAA model mice and 16 controls mice were obtained. One group mice model (*n*=6) and control (*n*=6) were killed. The aorta was removed and fixated in RNA later for 24 h thereafter frozen in −20°C and blood was taken to extract serum.

In order to study the significance of miR-155 inhibitors in AAA development, the rest of AAA model (*n*=30) mice were divided into three groups (10 mice every group). Groups were divided as follows: miR-155 antagomir group, antagomir NC group (group were treated with the Meaningless fragments), AngII (without treatment), and a saline treatment group was used as the control (*n*=10). miR-155 antagomir and antagomir NC (Ambion; Austin, TX) were injected into the abdominal aorta of AAA model mice for twice a week. On the 7th day, aneurysm specimens of mice were quickly collected and frozen to −80°C until analysis.

### Macrophage isolation

Peripheral blood mononuclear cells (PBMCs) from the blood (15 ml) of patients with AAA (*n*=11) and normal volunteer (*n*=15) using Lymphocyte Separation Medium (Haoyang Biological Manufacture Co., Tianjin, China). Briefly, the heparinized blood was diluted with phosphate buffered saline in proportion of 1:1, then layered over lymphocyte separation medium (1:1). PBMCs were obtained by centrifugation [36], and incubated with mouse-anti-human CD68 (1:400; Dako, Glostrup, Denmark) and goat anti-mouse IgG conjugated magnetic beads (Dynal, Oslo, Norway) according to the manufacturer’s instructions. CD68+ cells were isolated using flow cytometry (BD Biosciences, San Jose, CA, U.S.A.). The cell suspension was cultured in α-minimum essential medium (Αmem, Gibco, Invitrogen, Waltham, MA, U.S.A.) supplemented with 10% fetal bovine serum (PAA, Basel, Switzerland), 100 μM P/S (Sigma-Aldrich), and 25  ng/ml monocyte colony-stimulating factor (M-CSF, Peprotech, London, U.K.).

### Cell culture and miRNA mimic and inhibitor transfection

The human cell line THP-1 and human vascular smooth muscle cells (HVSMCs) were purchased from ATCC (American Type Culture Collection, Nr. TIB-202, Wesel, Germany). The human cell line THP-1 was cultured in RPMI-1640 media (Thermo Scientific, Waltham, U.S.A.) supplemented with 10% fetal bovine serum (PAA) and 100 μM P/S (Sigma-Aldrich). Cells were maintained at densities between 0.5 and 1.0 × 10^6^cells/ml in culture. HVSMCs were grown in medium DMEM/F12 (Gibco. Grand Island, NY) supplemented with 20% fetal bovine serum (PAA) and 100 μM P/S (Sigma-Aldrich). Cells were incubated at 37˚C and 5% CO_2_–95% air.

The miR-155 mimic (Thermo Fisher, Waltham, MA, U.S.A.) and miR-155 inhibitor (Exiqon Inc, Woburn, MA, U.S.A.) were transfected into THP-1 cells by using X-treme GENE siRNA transfection reagent (Mirus Bio LLC, Madison, WI, U.S.A.). Cells were transfected with nonsense sequence as controls (mimic NC and inhibitor NC). Inhibitor sequence were as follows: nonsense control (inhibitor NC) GTGTAACACGTCTATACGCCCA (Exiqon; 199020-00) and miR-155 inhibitor sequences were GTGTAACACGTCTATACGCCCA (Exiqon; 428232-00). miR-155 mimic sequences were UUAAUGCUAAUUGUGAUAGGGGU/AM17100 and miR control sequences were AM17111. THP-1 cells were transfected for approximately 48 h before transfection reagents were removed.

### Transwell

The migration assay of VSMC was performed in a transwell culture system, using a 5 μm membrane pore size (Transwell, Corning Costar, NY, U.S.A.), and 24-well culture plate. Briefly, 5 × 10^4^ VSMCs were seeded into the upper chamber containing 200 μl of non-serum DMEM medium, put into a 24-well plate filled with 600 μl of DMEM containing 10% FBS and 50% macrophage culture supernatants, and incubated for 24 h. Cells were fixed with 4% paraformaldehyde, and cells on the upper side of chamber were removed using a cotton swab. Then, cells on the lower side of chamber were stained with 0.1% Crystal Violet for 10 min. The cells that through the chamber were counted under a microscope and five random images were selected to quantify for each chamber.

### RNA isolation and qRT-PCR analysis

Total miRNA was extracted from AAA body, AAA neck, and serum of human or animal model of AAA or isolated macrophage using the miRVana miRNA Isolation kit (Ambion, Austin, TX, U.S.A.) and miRNeasy Serum/Plasma kit (Qiagen, Hilden, Germany) according to manufacturer’s specifications. miRNA concentration was quantified using a Nanodrop Spectrophotometer (Molecular Devices, Corp., Downingtown, PA). cDNA was synthesized by a TaqMan miRNA reverse transcription kit (Applied Biosystems, CA, U.S.A.) as per manufacturer’s instructions. The following primers were used in the PCR experiments: Human miR-155, forward primer: 5′-CGGTTTAATGCTAATCGTGA-3′, reverse primer: 5′-GAGCAGGGTCCGAGGT-3′; U6, forward primer: 5′-CTCGCTTCGGCAGCACA-3′, reverse primer: 5′-AACGCTTCACGAATTTGCGT-3′. Mouse miR-155, forward primer: 5′-AATGCTAATTGTGATAGGGGT-3′, reverse primer was provided in the kit; U6, forward primer: 5′-CTCGCTTCGGCAGCACA-3′, reverse primer: 5′-AACGCTTCACGAATTTGCGT-3′. RT-PCR was performed using the SYBR Green PCR core Reagent kit (Applied Biosystems, CA, U.S.A.). Reaction mixture was run in a 7500 Fast Real-Time PCR System (Applied Biosystems, U.S.A.) with denaturation step at 95°C for 10 min, followed by 45 cycles of denaturation at 95°C for 10 s and primer annealing/extension at 60°C for 60 s. Relative quantification of the target gene was analyzed using the comparative *C*_t_ (ΔΔ*C*_T_) method and normalized to U6 expression [37,38].

### Western blotting

Proteins were extracted from THP-1 transfected with miR-155 mimic or miR-155 inhibitor; HVSMC treated with supernatant of the transfection cells as above and tumor tissue from mouse. Western blotting was performed to assess the rabbit anti-MMP2 antibody (1:1000; Thermo Scientific, U.S.A.), goat anti-MMP9 antibody (1:500; Santa Cruz Biotechnology, U.S.A.), rabbit anti-monocyte chemoattractant protein (MCP-1) antibody (1:500; Bio-Rad Laboratories, U.S.A.), anti-iNOS antibody (1:100, Santa Cruz), anti-TNFα (1:1000, Sigma-Aldrich), anti-α-SMA antibody (1:1000; Millipore U.S.A.), and anti-Osteopontin antibody (1:500; Millipore, U.S.A.). Briefly, proteins (30 µg) were separated using 12% SDS gel electrophoresis and electrotransferred onto the polyvinylidene difluoride (PVDF) membrane (Millipore, Bedford, MA, U.S.A.). Then membranes were blocked with 5% nonfat dry milk in TBS-Tween for 1 h at room temperature, and incubated with primary antibodies at 4°C overnight. Horseradish peroxidase (HRP)-conjugated secondary antibody was used at a 1:5000 dilution (DAKO) for 2 h at room temperature. Bands were visualized using enhanced chemiluminescence (ECL Advance™; GE Healthcare). Protein expression was quantified with densitometric analysis using the ChemiDoc™ imaging system (Bio-Rad Laboratories) and QuantityOne™1-D Analysis Software (Bio-Rad Laboratories).

### Immunostaining

Histology and immunohistochemistry were performed on aneurysm tissues from patients and mice with AAA model. Paraffin sections (3 μm) were deparaffinized, rehydrated, and stained with hematoxylin and eosin (HE) (Sigma-Aldrich, St. Louis, U.S.A.) and Verhoeff’s Van Gieson (EVG; Aspen Biotechnology, China) staining according to the manufactures’ guidelines. For immunohistochemistry, sections were dewaxed, rehydrated and repaired, then were treated with 3% H_2_O_2_/0.1% sodium azide/PBS, Endogenous avidin, and biotin were blocked with endogenous avidin and biotin block kit (Vector Laboratories). Biotinylated rabbit anti-mouse IgG (1:100; Vector Laboratories) and goat anti-rabbit horseradish-peroxidase-conjugated IgG (1:500; DakoCytomation) were used to detect anti-CD 68 (1:100; BOSTER) antibody. Sections were incubated with the peroxidase substrate 3,3′-diamminobenzidine (ImmPACT DAB, Vector) for 1–2 min, counterstained in hematoxylin (ProSciTech), dehydrated, cleared in xylene, and mounted in entellan mounting medium (Electron Microscopy Sciences, U.S.A.). HVSMCs were stimulated with 5% culture supernatant of THP-1 cells transfected with miR-155 mimi and miR-155 inhibitor for 12 h. Cells were stained using PCNA (1:500, GeneTex, U.S.A.) overnight at 4°C, then washed with PBS and incubated with the corresponding secondary antibody for 30 min at room temperature. The cells were washed with PBS and incubated using DAB for 1 min. Pictures were produced using a Nikon Eclipse 50i microscope (Nikon, Japan).

### ELISA

The serum was isolated from patients with AAA by centrifugation. IL-1β, TNFα, and IL-6 levels in serum of patients with AAA or supernatant of THP-1 cells were detected using an ELISA kit (all purchased from Sigma–Aldrich) following the manufacturer's protocol.

### Statistical analysis

Statistical significance was determined using independent-samples *T* test or analysis of variance (ANOVA) followed by Tukey’s post-hoc test. *P*<0.05 was considered statistically significant. Analysis was completed with the GraphPad Prism.

## Results

### High expression of miR-155 and cytokines in patients with AAA and mouse models

**Figure 1:**
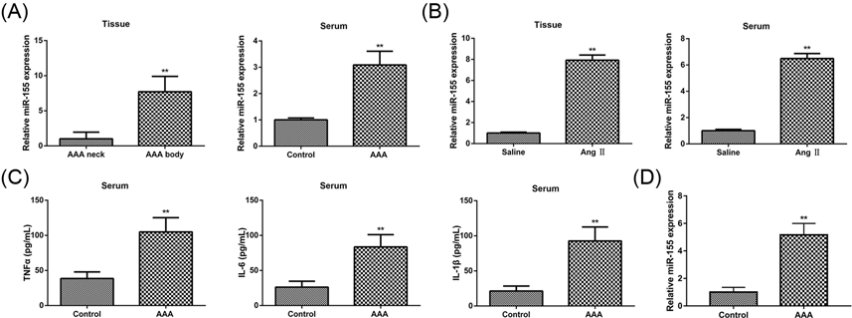
The expression levels of miR-155 (**A**) QRT-PCR analysis of miR155 expression levels in patients with AAA (*n*=11), (**B**) in AAA model mice (*n*=10). (**C**) Analysis of serum IL-1, IL-6, and TNFα level in patients with AAA (*n*=11) and control (*n*=15) by ELISA. (D) QRT-PCR analysis of miR-155 levels in macrophage from peripheral blood of patients with AAA (*n*=11) and control (*n*=15). Data were presented as mean ± standard error; **P<0.01; AAA, abdominal aortic aneurysm; AAA body, maximum AAA dilatation; AAA neck, the macroscopically nondilated; AngII, AAA model mice; saline, control mice.

To provide an experimental proof of a direct interaction between miR-155 and the AAA, we performed quantitative reverse transcription polymerase chain reaction (qRT-PCR). The results showed that miR-155 level in patients with AAA was markedly increased compared with that in corresponding control (in body tissue and serum) (*P*<0.01) (Figure 1A). The results in the AngII–infused ApoE-/-mice were consistent with in patients with AAA (Figure 1B). Cytokines play a role in the progression of inflammatory diseases [39]. Next, ELISA was performed to assess the effect of AAA on inflammation in serum of AngII–infused ApoE-/-mice. The results showed that TNFα, IL-6, and IL-1β levels were significantly increased as compared with the control groups (*P*<0.01) (Figure 1C). In addition, miR-155 level in macrophage from peripheral blood of patients with AAA had a dramatical increase than that in control (*P*<0.01) (Figure 1D). These results indicated that that miR-155 is closely related to inflammation and tumor *in vivo*.

### Overexpression of miR155 promoted macrophage inflammasome activation and cytokines expression

**Figure 2:**
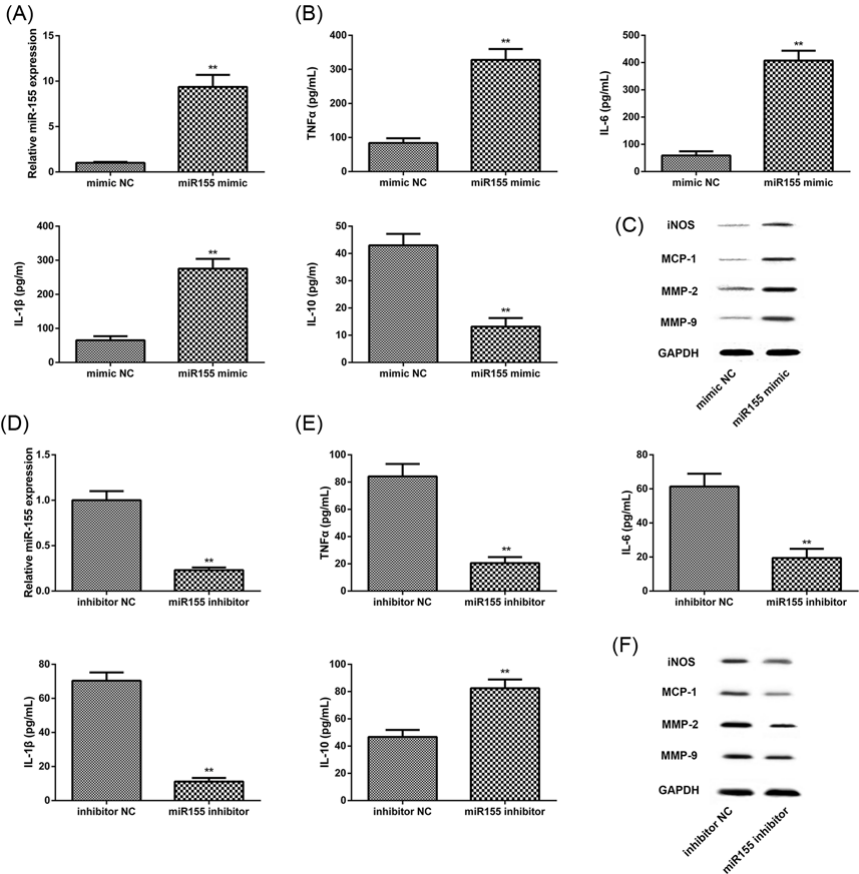
miR-155 exacerbated macrophage inflammasome activation *in vitro* (**A** and **B**) QRT-PCR analysis of miR-155 and cytokines levels with supernatant of miR-155 mimic-transfered macrophage. (**C**) Western blot analysis of macrophage inflammatory protein level in transfected cells of miR-155 mimic and inhibitor NC. (**D** and** E**) QRT-PCR analysis of miR-155 and cytokines levels in supernatant of miR-155 inhibitor-transfered macrophage. (**F**) Western blot analysis of macrophage inflammatory protein level in transfected cells of miR-155 inhibitor and inhibitor NC. Each experiment was repeated three times with three replications. Data are mean ± SEM from two independent experiments, **P<0.01.

Our previous works have shown an increased miR-155 expression in AAA biopsies and serum. Meanwhile, expression of the IL-1, IL-6, and TNFα were up-regulated. To investigate whether AAA is closely related to the regulation of miR-155 upon inflammatory stimulus, the human THP-1 cells were transfected with the miR-155 mimic, miR-155 inhibitor, and their negative controls. We first detected the level of miR-155 expression in THP-1 cells transfected with the miR-155 mimic, miR-155 inhibitor. As shown in Figure 2A,D, the expression of miR-155 was increased in transfection cells of miR-155 mimic, but decreased in transfection cells of miR-155 inhibitor, reflecting a high transfection efficiency.

Next, inflammatory cytokines in culture medium and surface proteins of macrophages from cells were measured by ELISA and Western blot. The result showed that up-regulation of miR-155 promotes the IL-6, IL-1β, and TNFα expression (Figure 2B), but IL-10 as an anti-inflammatory factor was lowered in cells that were transfected with miR-155 mimic (Figure 2B). Besides, we found that overexpression of miR-155 promoted MMP-2, MMP-9, iNOS, and MCP-1 protein expression in THP-1 macrophages (Figure 2C). While inhibition of miR-155 had an opposite effect (Figure 2E,F).

### miR-155 mimic promoted the proliferation and migration of VSMC by regulating macrophage-induced inflammation

**Figure 3:**
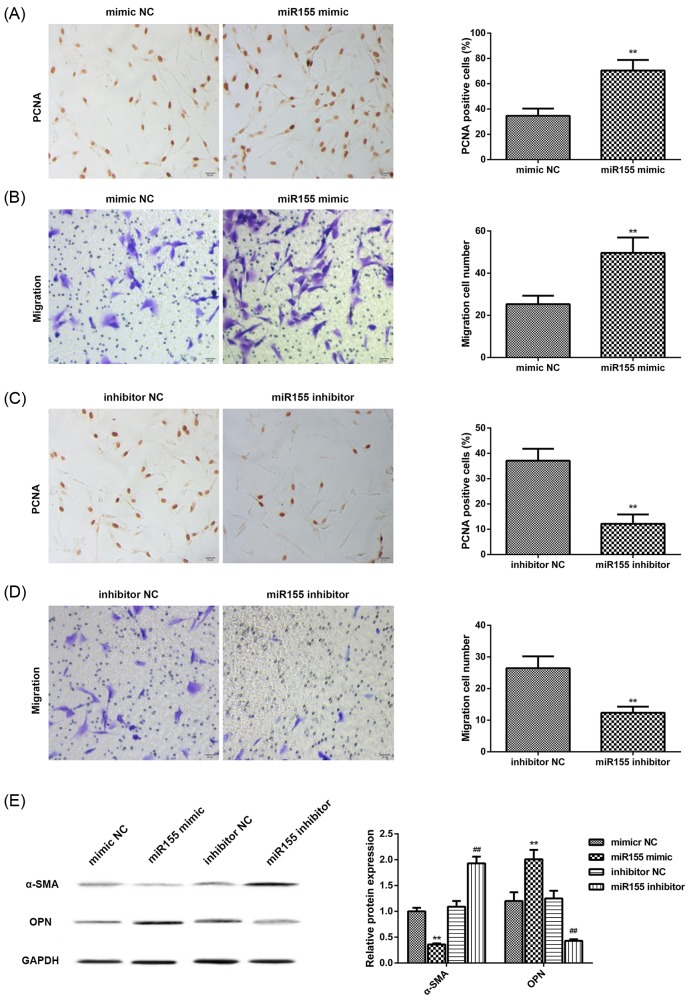
Macrophage-induced migration and proliferation of VSMC (**A** and **B**) Representative images of proliferation and migration of VSMC in miR-155 mimic group and mimic NC group. (**C** and **D**) Representative images of proliferation and migration in miR-155 inhibitor group and inhibitor NC group; scale bar = 20 µm; **P<0.01. *P*-values were calculated by independent samples *T* test. On the right is quantification of the proliferation and migration of VSMC. (**E**) Expression of α-SMA and OPN protein in miR-155 mimic group and miR-155 inhibitor group. On the right is quantification of α-SMA and OPN protein. Each experiment was repeated three times with three replications. *P*-values were calculated by ANOVA with Tukey’s post-test; **P<0.01, ^##^*P*<0.01.

Activated VSMC undergo dedifferentiation leading to reduction in contractile and cytoskeletal gene expression, but rise in expression of genes involved in the proliferation, migration, and matrix remodeling [40,41]. We further explored whether macrophage has a marked effects on VSMC proliferation and migration when miR-155 overexpression. Immunostaining and transwell assay were used to determine VSMC proliferation and migration. The result showed that proliferation and migration of VSMC treated with supernatant of macrophage transfected with miR-155 mimic were significantly increased compared with control (mimic NC) (Figure 3A,B). However, the treatment with supernatant from macrophage transfected with miR-155 inhibitor showed a significant reduction compared with control group (inhibitor NC) (Figure 3C,D). In addition, the result of Western blotting showed that expression of the OPN (a marker of proliferative VSMC) was increased and α-SMA (a marker of Systolic VSMC) was decrease in VSMC cultured with miR-155 mimic-transfectied macrophage supernatant (Figure 3E). However, the inhibitor treatment group showed an opposite results (Figure 3E).

### Down-regulation of miR-155 could prevent the development of AAA

**Figure 4:**
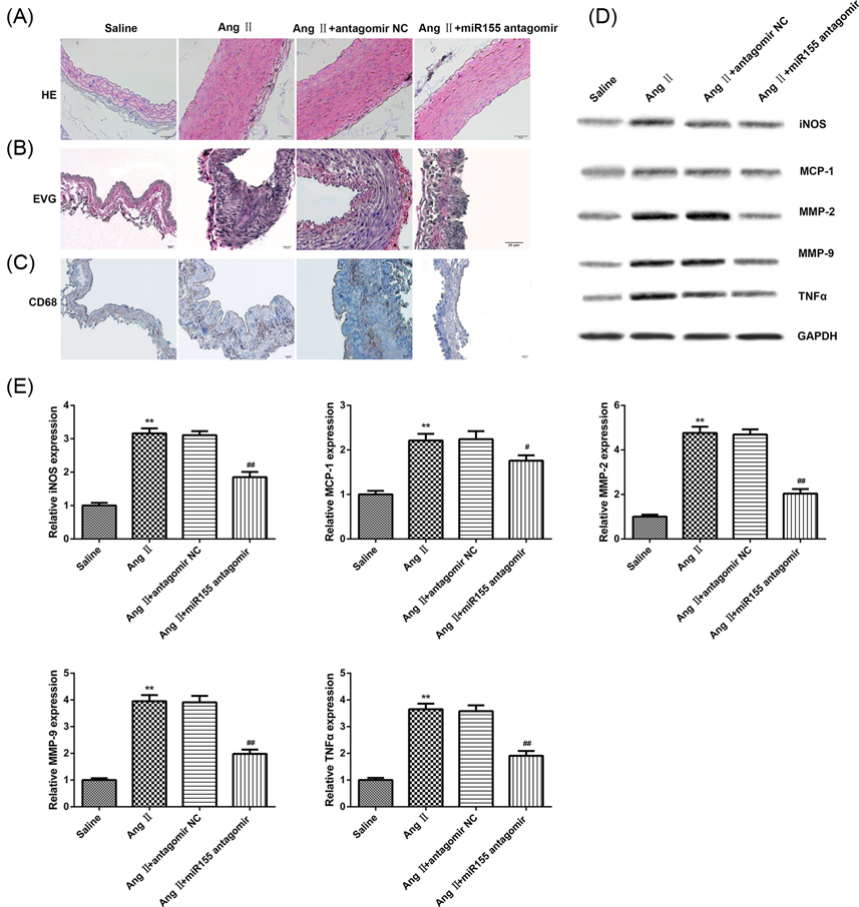
miR-155 antagomir can block macrophage inflammasome ameliorated severity of AAA (**A**) Representative images of HE staining; scale bar = 20 µm. (**B**) Representative images of EVG staining for elastic fiber collagen; scale bar = 20 µm. (**C**) Representative images of immunohistochemical staining for CD68+ macrophages; scale bar = 20 µm. (**D**) Western blot analysis of MMP-2, MMP-9, MCP-1, and TNFα in tissue of mice injected with miR-155 antagomir (*n*=10) and control (*n*=10). (**E**) Quantification of MMP-2, MMP-9, MCP-1, and TNFα. *P*-values were calculated by ANOVA with Tukey’s post-test; **P<0.01; ^##^*P*<0.01.

It has been established that miR-155 is able to cause chronic inflammation, and macrophages recruited may promote MMP-2 and MMP-9 expression to lead to the destruction of collagen fibers around artery vascular [42–44]. So, it is speculated that miR-155 is associated with the inflammation mediated by macrophage in the AAA. To investigate the effect of macrophage on AAA formation *in vivo*, we observed the morphological change in AAA model mice. The histological staining showed that miR-155 antagomir significantly inhibited the vasculopathy compared with antagomir NC and AngII group (Figure 4A). Verhoeff’s Van Gieson (EVG) staining showed that elastic collagen-positive areas were significantly decreased in the tissue of the miR-155 antagomir injection group compared with that in antagomir NC (Figure 4B). In addition, we found that CD68 was observably decreased in the tissue of the miR-155 antagomir injection group than that in antagomir NC group (Figure 4C). Furthermore, the results of Western blotting showed that miR-155 antagomir treatment inhibited the expression of MMP-2, MMP-9, MCP-1, and TNFα protein (Figure 4D,E). These results suggested that macrophage level was increased in AAA-ruptured regions and involved in elastic collagen fiber destruction, leading to an increased risk of AAA.

## Discussion

AAA is a serious late onset vascular disease with high mortality. miR-155 may play a role in AAA pathology based on influence of its overexpression and deficiency exist on inflammatory factors [45,46]. However, the specific regulation mechanisms of the miR-155 remain unknown and further studies are required. Our studies have shown that the miR-155 expression in human AAA aortic wall tissue and serum was quite different from that in normal human. miR-155 is closely related to inflammation and tumor [47]. In our research, miR-155 promoted expression of macrophage and cytokines (TNFα, IL-6, and IL-1β) in AAA. Nowadays, there is no specific test available for monitoring of AAA. Given the miR-155 and association with AAA, they have been suggested as potential diagnostic biomarker [48].

Macrophage is one of the critical cells of inflammation and immunology, and the expression of miR-155 is increased under various inflammatory transmitters [8,49]. The existence of macrophage can injure the arterial wall because the collagen production are disintegrated by MMP product from macrophage [29]. The present study is that miR-155 level correlated with AAA dilation in AngII-induced AAA of mice. This was consistent with previous findings in human AAA. Because it has been established that miR-155 is able to cause chronic inflammation, it is speculated that macrophage is associated with the inflammations in the AAA [50,51]. Namely, miR-155 enhances expression of MMP from macrophage, subsequently, which leads to macrophage infiltration [52]. Indeed, we also found the level of inflammation was significantly increased in serum from patients with AAA and cellular supernatant of THP-1 cells transfected with miR-155 mimic, indicating overexpression of miR-155 promoted aortic elastin degradation and destruction, which led to increased susceptibility of AAA. Besides, migration of VSMCs leads to aortic aneurysms [53,54]. Our study showed that expression of the OPN was increased and α-SMA was decreased when VSMC was treated with supernatant of miR-155 mimic-transfected macrophage [55]. These results indicate inhibiting inflammation to prevent the appearance of arterial wall rupture in AAA.

The increased number of inflammatory cytokine and recruited macrophages may cause an increase in MMP-2 and MMP-9 levels leading to the destruction of collagen fibers around artery vascula [56–58]. In our result, Verhoeff’s Van Gieson (EVG) staining and Western blot showed that elastic collagen-positive areas, the protein levels of MMP-2, MMP-9, MCP-1, TNFα, and macrophage infiltration significantly decreased in the tissue of the miR-155 antagomir injection group. These results suggest that suppression of miR-155 could attenuate infiltration of macrophage in AAA. Present studies indicate that the miR-155 is associated with cytokines and macrophage resulting in the increased AAA rupture risk [59]. Appropriate control of miR-155 by regulating macrophage in the vascular wall may be an important strategy to prevent AAA rupture.

In conclusion, our studies indicate that inhibition of miR-155 could attenuate inflammatory response and matrix protein hydrolysis in AAA by regulating macrophage. miR-155 play a critical role in controlling macrophage phenotype, understanding the differentiation and effector functions of macrophage regulated by miR-155 may provide a novel intervention target for AAA.

## Abbreviations

α-SMA: α-smooth muscle actin
AAA: abdominal aortic aneurysms
AngII: Angiotensin II
IL-1β: interleukin-1 beta
IL-6: interleukin-6
iNOS: inducible nitric oxide synthase
MCP-1: monocyte chemoattractant protein-1
MMP-2: matrix metalloproteinase-2
MMP-9: matrix metalloproteinase-9
PBMC: peripheral blood mononuclear cell
qRT-PCR: quantitative reverse transcription polymerase chain reaction
TNFα: tumor necrosis factor-alpha
VSMC: vascular smooth muscle cell
